# 105. Expanding and Sustaining Influenza Vaccination Acceptance amongst Adults with HIV in Delaware during the COVID-19 Pandemic

**DOI:** 10.1093/ofid/ofac492.183

**Published:** 2022-12-15

**Authors:** Deborah A Kahal, Christopher James, Brian Wharton

**Affiliations:** ChristianaCare/Thomas Jefferson University, Media, Pennsylvania; ChristianaCare, Wilmington, Delaware; ChristianaCare, Wilmington, Delaware

## Abstract

**Background:**

Influenza vaccination remains vital for people with HIV (PWH) who experience more severe disease and poorer outcomes than people without HIV. During the ongoing COVID-19 pandemic, influenza vaccination indirectly eases strain on healthcare systems. Historical annual adult influenza vaccination rates were 65% with 85.5% coverage achieved during a 2020-21 pilot site (N=750) quality improvement (QI) project.

**Methods:**

An expanded QI initiative sought to again achieve ≥80% influenza vaccination coverage for the 2021-22 season in PWH ≥18 years old attending program sites throughout Delaware (N=1853) (Fig. 1). A voluntary multidisciplinary team continued the initiative with a fishbone diagram detailing previously successful, rapidly implementable, and reproducible levers for change (Fig. 2). Space changes included: diverse, prominent visual displays, phone messaging, and virtual platform use. The team provided consistent, multi-modality staff education. Vaccinations were confirmed by statewide electronic health records or tangible proof-of-vaccination receipt and cross-referenced with Ryan White CareWare data. Overall and county-specific vaccination rates were monitored and displayed weekly to patients and staff.

**Figure:**
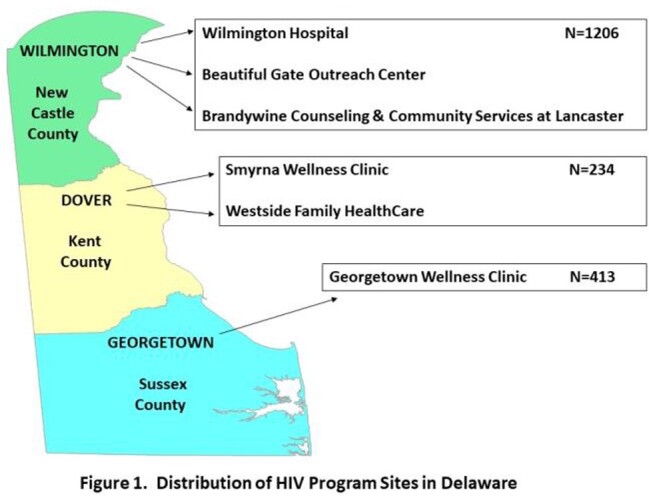

**Figure:**
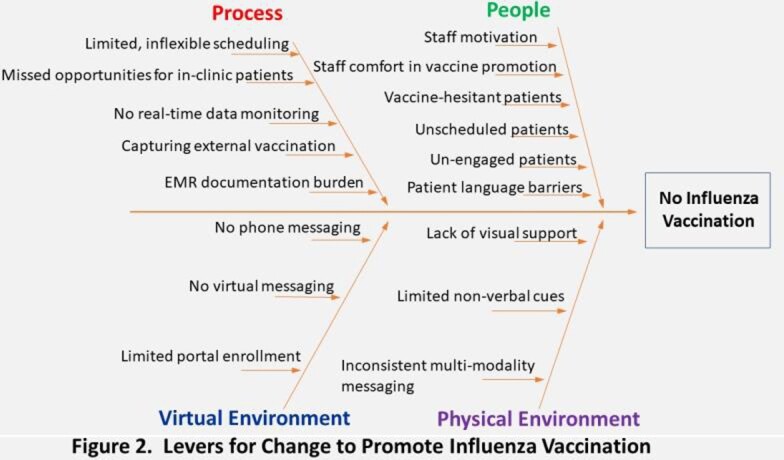

**Results:**

84.9% vaccination coverage was achieved program-wide with county level rates ranging from 79.1 to 89.7% (Table 1).

**Figure:**
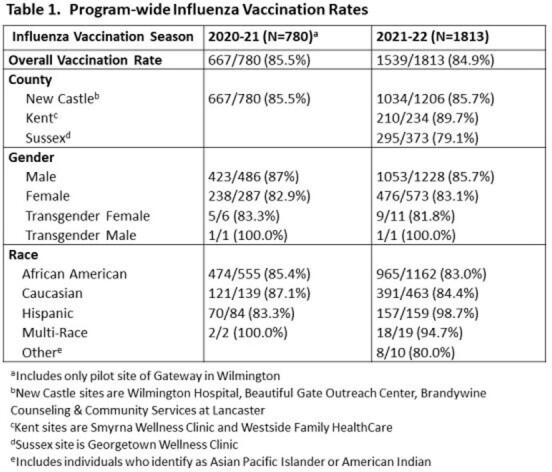

**Conclusion:**

Our statewide initiative to sustain and expand high influenza vaccination rates again exceeded 80% in adult PWH attending an array of HIV clinics during the COVID-19 pandemic. Components vital to success include harnessing a motivated multidisciplinary team, focusing on feasible, wide-reaching change levers to promote desirability and accessibility of vaccination to patients, and real-time sharing of progress to patients and staff. The bundled approach challenges efforts to understand individual change effects on outcomes. Opportunities to improve site-specific outcomes remain. Translation of this work has contributed to >85% of PWH receiving ≥1 COVID-19 vaccination, with a goal of further program-wide expansion targeting all administered vaccinations.

**Disclosures:**

**All Authors**: No reported disclosures.

